# Host Gene Expression Profiling of Dengue Virus Infection in Cell Lines and Patients

**DOI:** 10.1371/journal.pntd.0000086

**Published:** 2007-11-21

**Authors:** Joshua Fink, Feng Gu, Ling Ling, Thomas Tolfvenstam, Farzad Olfat, Keh Chuang Chin, Pauline Aw, Joshy George, Vladimir A. Kuznetsov, Mark Schreiber, Subhash G. Vasudevan, Martin L. Hibberd

**Affiliations:** 1 Novartis Institute for Tropical Diseases, Chromos, Singapore; 2 Genome Institute of Singapore, Genome, Singapore; Oxford University Clinical Research Unit

## Abstract

**Background:**

Despite the seriousness of dengue-related disease, with an estimated 50–100 million cases of dengue fever and 250,000–500,000 cases of dengue hemorrhagic fever/dengue shock syndrome each year, a clear understanding of dengue pathogenesis remains elusive. Because of the lack of a disease model in animals and the complex immune interaction in dengue infection, the study of host response and immunopathogenesis is difficult. The development of genomics technology, microarray and high throughput quantitative PCR have allowed researchers to study gene expression changes on a much broader scale. We therefore used this approach to investigate the host response in dengue virus-infected cell lines and in patients developing dengue fever.

**Methodology/Principal Findings:**

Using microarray and high throughput quantitative PCR method to monitor the host response to dengue viral replication in cell line infection models and in dengue patient blood samples, we identified differentially expressed genes along three major pathways; NF-κB initiated immune responses, type I interferon (IFN) and the ubiquitin proteasome pathway. Among the most highly upregulated genes were the chemokines IP-10 and I-TAC, both ligands of the CXCR3 receptor. Increased expression of IP-10 and I-TAC in the peripheral blood of ten patients at the early onset of fever was confirmed by ELISA. A highly upregulated gene in the IFN pathway, viperin, was overexpressed in A549 cells resulting in a significant reduction in viral replication. The upregulation of genes in the ubiquitin-proteasome pathway prompted the testing of proteasome inhibitors MG-132 and ALLN, both of which reduced viral replication.

**Conclusion/Significance:**

Unbiased gene expression analysis has identified new host genes associated with dengue infection, which we have validated in functional studies. We showed that some parts of the host response can be used as potential biomarkers for the disease while others can be used to control dengue viral replication, thus representing viable targets for drug therapy.

## Author Summary

Dengue is the most prevalent mosquito-born viral disease affecting humans, yet there is, at present, no drug treatment for the disease nor are there any validated host targets for therapeutic intervention. Using microarray technology to monitor the response of virtually every human gene, we aimed to identify the ways in which humans interact with dengue virus during infection in order to discover new therapeutic targets that could be exploited to control viral replication. From the activated genes, we identified three pathways common to *in vitro* and *in vivo* infection; the NF-κB initiated immune pathway, the type I interferon pathway, and the ubiquitin proteasome pathway. We next found that inhibiting the ubiquitin proteasome pathway, or activating the type I interferon pathway, resulted in significant inhibition of viral replication. However, inhibiting the NF-κB initiated immune pathway had no effect on viral replication. We suggest that drugs that target the ubiquitin proteasome pathway may prove effective at killing the dengue virus, and, if used therapeutically, improve clinical outcome in dengue disease.

## Introduction

Although dengue-related disease results in an estimated 50–100 million cases of dengue fever and 250,000 to 500,000 cases of dengue hemorrhagic fever/dengue shock syndrome each year [1],[2], a clear understanding of dengue pathogenesis remains elusive. Dengue virus is an enveloped, positive-stranded RNA virus of the *Flaviviridae* family transmitted by the mosquito *Aedes aegypti* and *Aedes albopictus*. Four serotypes of dengue virus (DENV1-4) circulate in endemic areas. Although infection with one serotype of dengue virus confers life-long protective immunity to that serotype, it does not protect the host from infection with other serotypes [3].

The initial target cells during dengue infection are believed to be Langerhans cells [4]. Through means not yet fully understood, Langerhans cells spread the virus, via the lymphatic system, to other tissues such as liver, spleen, kidney and blood, whereas monocytes, macrophages and endothelial cells are the major cell types in which the virus replicates [5]. In the majority of symptomatic dengue infections, a fever of 5–7 days duration develops together with bone and joint pain, retro-orbital pain, nausea and fatigue, this is called dengue fever (DF). While the majority of DF patients recover without intervention, 2–5% develop a more severe form of the disease, called dengue hemorrhagic fever/dengue shock syndrome (DHF/DSS), characterized by thrombocytopenia and vascular leakage, causing hypervolemic shock and death if not promptly treated [6]. The cause of DHF/DSS is not clear. Antibody dependent enhancement (ADE) is the most widely supported theory explaining the higher risk of DHF/DSS associated with a heterologous secondary infection [7]. The phenomenon of T cell “original antigenic sin” has also been described [8]. Like many viruses, dengue inhibits IFNα and IFNβ signaling by suppressing Jak-Stat activation, resulting in reduced host antiviral response [9]. The combination of a reduced host defense, increased uptake of the virus and delayed viral clearance likely synergizes to produce higher viremia resulting in a more severe outcome.

Because of the lack of a disease model in animals and the complex immune interaction in dengue infection, the study of host response and immunopathogenesis is difficult. The development of genomics technology has allowed researchers to study gene expression changes on a much larger scale. A summary of published microarray data from infection of different host cell types with bacteria, viruses, yeast, protozoa and helminthes revealed a common host-transcriptional-response consisting of a cluster of IFN-stimulated and immune mediating genes [10]. One particularly successful application of microarray technology was the identification of a novel drug target, c-kit, in endothelial cells infected with Kaposi's sarcoma-associated herpesvirus (KSHV) [11].

With regard to dengue infection, gene expression studies have been carried out in infected human umbilical vein endothelial cells (HUVECs) by differential display reverse transcription (DD-RTPCR) and Affymetrix oligonucleotide microarrays [12]. Genes having a role in the IFN antiviral response and immune defense such as 2′–5′ oligoadenylate synthetase (OAS), myxovirus protein A (MxA), TNFα, galectin-9, phospholipid scramblase 1 and human inhibitor of apoptosis-1 (IAP1) were shown to be upregulated upon infection [12]. Infection with dengue of a more transformed HUVEC-like cell line, ECV304, was analyzed by a different microarray system containing 7600 cDNA oligonucleotides [13]. In this study, the expression of 15 genes involved in cell cycle, apoptosis, membrane trafficking and cytoskeleton was found to be altered after infection[13]. Using a different gene expression approach, primary macrophages infected with a clinical isolate of dengue were analyzed by a cytokine array containing 375 human cytokine-related genes with approximately 20 genes observed to be either up- or down- regulated [14]. However, the functional importance of these changes, if any, was not studied [14].

We have used Compugen human 19K oligonucleotide arrays to study the host response to dengue infection, initially in a human hepatocytic cell line (HepG2). We identified the upregulation of three major clusters of genes; NF-κB-mediated cytokine/chemokine responses, type I IFN response, and the ubiquitin-proteasome system. Increased expression of selected genes, identified by Compugen array, was confirmed using a taqman low density array (TLDA) and results extended to include an additional, readily infectable, cell line (A549) and PBMC derived from adult dengue fever patients from a Singapore dengue prospective cohort study. Next we sought to confirm the functional effects of these upregulated genes. We confirmed the high levels of two novel chemokines, IP-10 and I-TAC, in dengue infection, in both cell lines and in dengue patients. We also determined that overexpression of viperin, an upregulated gene in the IFN pathway, and proteasome inhibition, with MG-132 or ALLN, reduced virus replication. In summary, we have used microarray analysis to identify new host genes associated with dengue infection, and to show that components of the host response can control dengue viral replication and therefore represent potential targets for drug therapy.

## Materials and Methods

### Cell lines and dengue virus

Cell lines, A549, BHK-21, C6/36, HeLa, HepG2, HUV-EC-C, K562, SK-Hep1 and THP-1, were obtained from ATCC and maintained as instructed. The type 2 dengue virus strain TSV01 was obtained from a dengue outbreak in Townsville, Australia [15] (GenBank, accession number AY037116) and was propagated in the C6/36 cell line. Heat-inactivated virus was prepared by incubating virus samples in a 55°C water bath for 1 hr. Each cell line was infected with dengue virus at a multiplicity of infection (MOI) of 1 and 10 for 3, 6, 12, 24, 48, and 72 hrs before being evaluated for replication efficiency by plaque assay. Infection of cell lines was not continued beyond 72 hrs as after this point a significant degree of cell death became apparent.

### Plaque assay

BHK-21 cells were cultured overnight in 24 well plates before media was removed and serial dilutions (10-fold) of virus culture supernatants added to individual wells. Plates were incubated for 1 hr before media was aspirated and replaced with 0.5 ml of 0.8% methyl-cellulose medium (with 2% FBS). Plates were then incubated for 5 days before the media was removed and cells fixed in 4% formaldehyde for 20 min, rinsed in water, stained with crystal violet for 20 min then rinsed again. Plaques were counted manually and concentrations of plaque forming units per ml (pfu/ml) in the cell culture supernatant calculated. For drug intervention studies, HepG2 cells were treated with compound, or DMSO, at the indicated concentration and time. While the culture supernatants were collected for plaque assay, cytotoxicity in HepG2 cells was monitored using fluorescein diacetate (FDA, Acros Organics, Belgium), 10 µg/ml added to cells for 25 min at room temperature, and measured using a fluorescent plate reader (485 nm excitation/535 nm emission).

### Flow cytometry

Dengue E-protein was assessed in suspension HepG2 cells by intracellular staining and fluorescence activated cell sorter (Becton Dickinson, USA) and an Alexa-647 conjugated (AlexaFluor conjugation kit, Invitrogen, USA) monoclonal antibody (4G2, ATCC). Cells were scraped and permeabilized using BD FACS Perm/Wash solution (Becton Dickinson, USA) and acquisition and analysis was performed using CellQuest software (Becton Dickinson, USA).

### Virus quantification by real time RT-PCR

RNA was extracted from infected cells using the RNeasy Mini Kit (Qiagen, Netherland). Dengue serogroup 2 virus TSV01 detection by TaqMan PCR was adapted to the ABI7900 real time instrument using previously published primers and probes [16]. Standard ABI conditions were used, incorporating primers at 900nM. Quantification was achieved by relating viral Ct value to the Ct value on a standard curve of a measured number of copies of a 750 bp section of the virus (forward: 5′-AAAGATCAGTGGCACTCGTTCC-3′ and reverse: 5′-GCAGGTCTAAGAACCATTGCCT-3′), cloned into pCR2.1-TOPO (Invitrogen, USA).

### Microarray

Human arrays of 19,800 60mer oligonucleotide probes (representing 18861 genes), designed by compugen and manufactured by Sigma-Genosys were used according to the standard protocol described for cDNA microarray [17]. Dye swap was performed for each sample, at every time point and a rigorous quality check was performed before an array was used for downstream analysis [18].

60 mer oligonucleotide probes were spotted onto poly-L-lysine-coated microscope slides using GeneMachines OmniGrid Microarray Spotter (USA). For fluorescence labeling of target cDNAs, 20 µg of total RNA from universal human reference (Strategene, USA) and experiment samples (RNA extracted from infected cells using Qiagen RNeasy Mini Kit ) were reverse transcribed in the presence of Cy3-dUTP and Cy5-dUTP (Amersham Biosciences, UK) using the Superscript reverse transcription kit (Invitrogen, USA). Labeled cDNA were pooled, concentrated, re-suspended in DIG EasyHyb (Roche, Switzerland) buffer and hybridized overnight (14-16h) in the MAUI Hybridization chamber (BioMicro, USA). The arrays were scanned using a GenePix 4000B Scanner (Axon Instruments, USA) to generate Tiff images. The images were analyzed by GenePix Pro 4.0 software (Axon Instruments, USA) to measure Cy3 and Cy5 fluorescence signals intensity and format data for data base deposition. The array data then underwent lowess normalization [19] available in an R package aroma to remove channel specific biases (R Development Core Team).

### Selection of differentially expressed genes from microarray data

Differentially expressed genes were selected using a procedure known as Significance Analysis of Microarrays (SAM) [20], described in brief below. The statistic used in SAM is given as 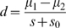 where; the numerator is the group mean difference, *s* the standard error, and *s*_0_ a regularizing constant. Setting *s*_0_ = 0 will yield a t-statistic. This value, called the fudge constant, is found by removing the trend in d as a function of *s* in moving windows across the data to reduce false positive results. As the statistic is not t-distributed, significance is computed using a permutation test. Genes with a computed statistic larger than the threshold were considered significant. The false discovery rate (FDR) associated with the given threshold can also be calculated from the permutation data.

### Quantitative PCR (qPCR) by TaqMan Low Density Array (TLDA)

RNA was extracted using RNase Easy kit (Qiagen, Netherland) from infected cells. For patients blood samples (see Patient Samples), 2.5 ml of blood was collected in PAXgene tubes, RNA was extracted using PAXgene Blood RNA Kit (PreAnalytiX, Qiagne, Netherland). RNA was subjected to DNase treatment using an RNase-Free DNase Set (Qiagen, Netherland). 100 ng of total RNA was reverse transcribed using the High-Capacity cDNA Archive Kit (ABI, USA) and processed for TaqMan Micro Fluidic Cards (3M Company, ABI, USA) according to manufacturers instructions, together with data analysis using SDS2.2 software (ABI, USA). Differentially expressed genes were detected as above, using SAM. We analyzed SAM gene lists using the Applied Biosystem online program PANTHER [21] (http://www.pantherdb.org/). Pathway, interaction and Gene Onotology analysis was performed using MetaCore, version 3.2.0 (GeneGo, Inc, USA).

### Patient samples

Subjects were enrolled from the Early DENgue (EDEN) study, a dengue investigation conducted at a primary healthcare clinic in Singapore, for which local ethical approval had been granted, and informed consent from patients obtained. Participation required a PCR diagnosis of fever with duration less than 3 days (for the details of the PCR diagnosis, see [22]). The enrolled dengue cases were either of serotype 1 or 3. Ten dengue positive subjects (n = 10, 3 males) were selected to represent the most severe cases of dengue by the criteria of a platelet counts below 30 (x 10^3^/µl; range 8–30, mean 20.5). Their mean age was 43.2 years (range 24–67). The mean fever was 38.3°C (range 37.7–39.1) at the first visit (Time Point 1), with a mean duration of 43 hours (range 14–72) from the onset of the fever. The second visit (Time Point 2) occurred at 80 to 96 hours after the first visit, mean fever was 37.1°C (range 36.2–38.7). Dengue negative patients were enrolled with the same criteria (fever with no respiratory infection symptoms), but were PCR negative for dengue. Ten subjects were taken with (n = 10, 4 males) a mean age of 43.2 years old (range 24–67). Their mean fever was 38.4°C (range 37.6–39.4) with a mean duration 27 hrs (range 8–60) at the first visit (Time Point 1). The convalescence sample was collected at the third visit which was 3 to 4 weeks later (Time Point 3). At each time point, a 2.5 ml blood sample was collected in PAXgene tubes for RNA analysis. A 10 ml blood sample was collected, serum was separated within 5 hrs and stored at −80°C. Serum IP-10 and I-TAC concentrations were measured using ELISA kits from R&D Systems as per manufacturers instructions.

### Generation of A549 cells stably expressing viperin and treatment with IFNβ

A549 cells were transfected with an expression construct encoding viperin using lipofectamine 2000 (Invitrogen, USA). Cells were selected using 500 µg/ml G418 and screened for viperin expression by immunoblotting. Cells were cultured overnight in 6 well plates before IFNβ (Glycoferon, Singapore), at a final concentration of 500 U/ml, was added to each well while control wells remained untreated. Twelve hours post IFNβ treatment, cells were infected with dengue virus (TSV01; MOI 1) for 48 hrs and the plaque assay was used to determine virus production.

## Results

### Establishing an infection model for dengue virus

While a number of different cell lines have been used as models for dengue infection it is not clear which of these represents the most appropriate model for the analysis of host response by microarray, which requires a high rate of infection. As such, we screened seven human cell lines for their ability to support replication of dengue virus. We used the clinical, dengue serotype 2 isolate TSV01 (Accession number: AY037116) strain for infection. Human cell lines were ranked by maximum plaque forming units (pfu)/ml titer produced with A549>HepG2>SK-Hep1>K562>HUV-EC-C>THP-1>HeLa (data not shown). The same results were obtained with the widely used NGC strain (data not shown). The highest yielding cell lines A549 (a lung carcinoma) and HepG2 (a hepatoma cell line) were used in further studies, with HepG2 as the primary focus because of evidence of liver injury in DHF/DSS and the detection of dengue antigens in hepatocytes in liver [23],[24].

### Microarray identification of host responses to dengue virus replication using the HepG2 infection model

**Figure 1:**
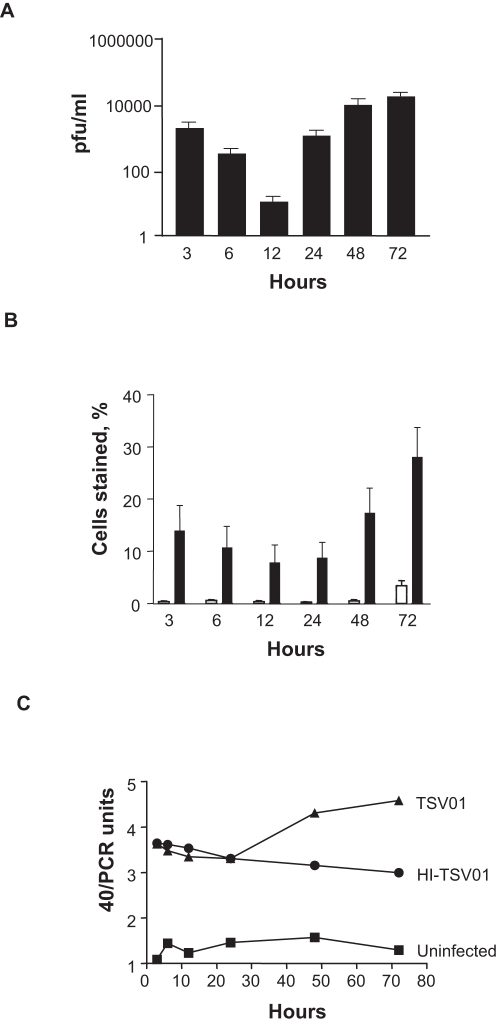
Dengue replication in HepG2 cells. (A) HepG2 cells were infected with dengue virus TSV01 (MOI 10) for 3, 6, 12, 24, 48 and 72 hrs. Cell culture supernatants were collected and assayed for dengue virus by plaque assay. Plaque forming units per ml are expressed on a log scale as mean±s.e.m. where n = 4. (B) HepG2 cells were infected with dengue virus TSV01 (closed bars) or incubated with media alone (open bars). Percentage of cells staining positive for dengue virus E-protein by FACS are expressed as mean±s.e.m. where n = 3. (C) HepG2 cells were infected with dengue virus TSV01 (MOI 10) for 3, 6, 12, 24, 48 and 72 hrs. RNA was extracted and used to quantitate viral RNA copies by Real Time PCR. The copy number is represented by dividing the cycle detection threshold (Ct) value at the limit of detection (40) by the sample Ct value (40/PCR units). One representative result is shown.

**Figure 2:**
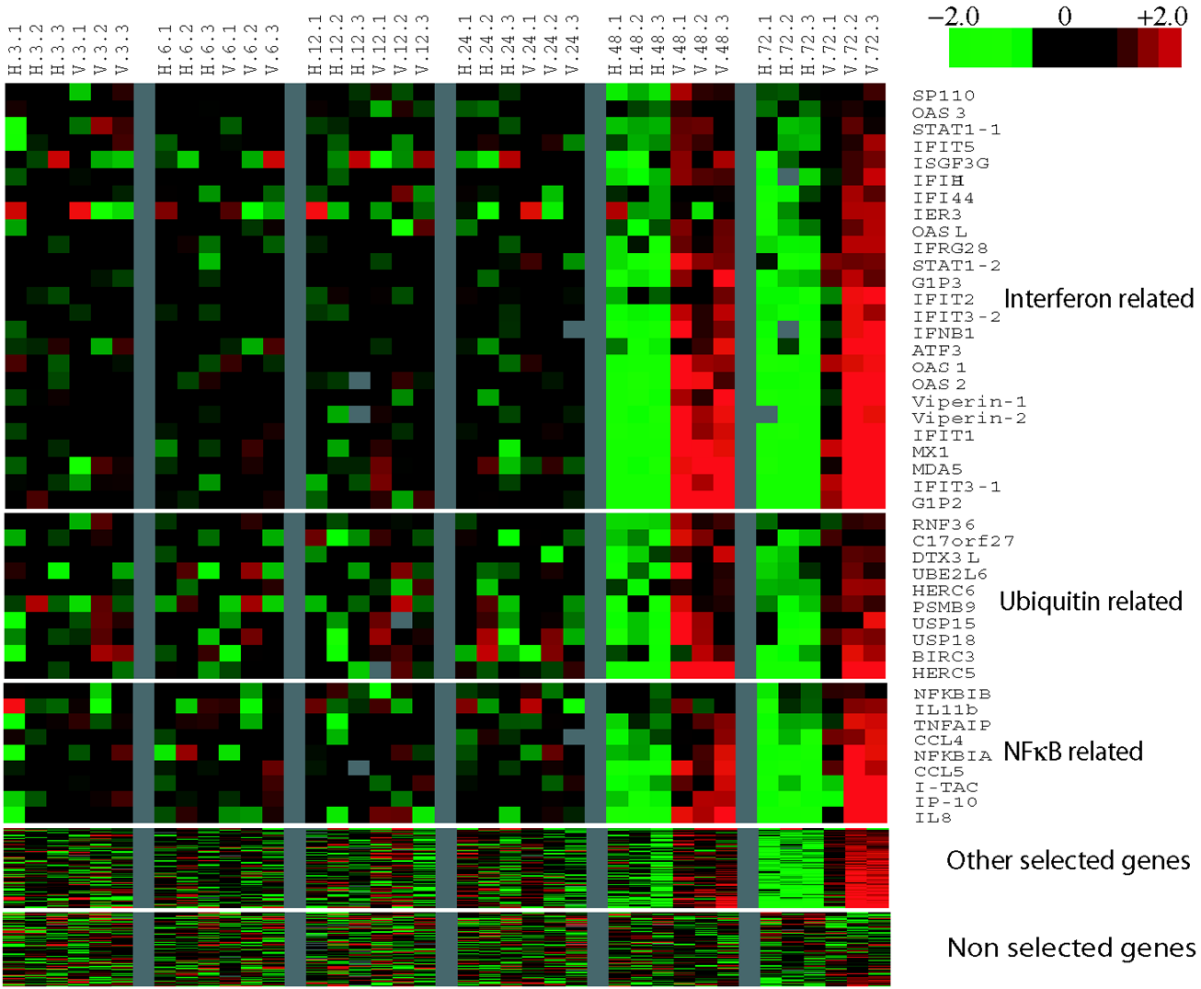
Heat map of the microarray gene expression profile of HepG2 cells treated with live dengue virus TSV01 (V) or heat inactivated TSV01 (H) transcripts identified as being differentially expressed by SAM analysis. Details on gene name, gene function, SAM statistics, and fold change for all 132 selected transcripts are given in Table S1. Colour intensity is derived from mean relative expression fold changes of dye swap results in comparison to universal reference (Strategene USA), red for upregulated, green for down regulated, black for no change, and grey for missing data. Time course (3, 6, 12, 24, 48, 72 hours post infection) and triplicate (.1, .2, .3) are indicated on the x axis and genes are grouped into three functional classes and clustered by expression profile on the y axis.

Viral replication in HepG2 cells infected with dengue virus TSV01 for 3, 6, 12, 24, 48 and 72 hours, compared to heat inactivated virus, was determined by plaque assay of the virus released in the cell culture medium (Figure 1A), FACS analysis of infected cells labeled intracellularly with an Alexa 674-conjugated antibody against dengue E protein (4G2) (Figure 1B) and real-time PCR analysis of viral RNA (Figure 1C). All three methods showed that new viral replication began after 24 hours and reached a plateau at 72 hrs, with FACS analysis showing 28% of cells infected at this point. After 72 hrs a degree of cell death become apparent (data not shown) and the experiment was not continued. Analysis of microarrays, performed in duplicate (dye-swapped) on three biological replicates at each time point, comparing infectious with heat inactivated virus; using a SAM q value (false discovery threshold) of 25%, revealed no significantly differentially expressed genes at 3, 6, 12 or 24 hrs post infection. However, there were 24 transcripts identified at 48 hrs and 124 at 72 hrs (a total of 132 transcripts representing 124 genes; Table S1) that were differentially expressed. At both 48 hrs and 72 hrs, clustering of transcripts using PANTHER analysis identified the IFN-mediated immunity pathway as the most significant with a P value of 10^−15 ^(at 72 hrs). Genes that are typically induced after type I IFN stimulation, including OAS1, OAS2, OAS3, OASL, STAT1, STAT2, MX1, IFIH and IFNβ, featured prominently in this cluster (Figure 2 and Table S1). Further analysis of the 124 genes suggested the involvement of NF-κB-mediated cytokine/chemokine responses (NFKBIB, NFKB1A, TNFA1P, CCL4, CCL5, IP-10 and I-TAC amongst others) and ubiquitin related genes (HERC5, HERC6, UBE2L6, USP15 and others) (Figure 2). Although not completely overlapping, a core host response to pathogen involving the IFN response and the NF-κB-mediated immune defense response [10] was observed in our array results. Other genes that were significantly changed included those associated with cell signaling, lipid metabolism, cell cycle and vesicular transport (Table S1). We did not detect any significantly down regulated genes in our system. All the microarray data were deposited in a public database accessible at http://www.ncbi.nlm.nih.gov/projects/geo (accession number is GSE6048).

### Confirmation and validation of upregulated genes by quantitative PCR in cell lines and in dengue patients

With the evidence of upregulation of three major pathways (NF-κB, IFN and ubiquitin) by microarray, we decided to pursue these pathways with further confirmation and validation. Firstly, we chose 59 genes from the three major pathways that were upregulated in the HepG2 cell line as shown by microarray. Secondly we selected another 36 genes which had a functional association with these pathways or might otherwise be associated with dengue infection. A Taqman Low Density Array (LDA) was then constructed for these 95 genes in order to confirm their expression by quantitative real time PCR. Results for the HepG2 cell line indicated that there was again no significant difference between infectious and heat-inactivated virus at the 24 hr time point post-infection. At 48 hrs 31 of the 95 genes were differentially expressed, and at 72 hrs 62 of the 95 genes were differentially expressed (Table S2), indicating a high rate of validation of the genes detected by microarray.

In order to exclude gene responses that were specific to the HepG2 cell line alone, we decided to test the expression level of these genes in the A549 cell line using the same LDA. In the A549 infection model, plaque assay revealed that peak viral production occurred earlier than in the HepG2 model (1.1×10^5^±1.4×10^4^ pfu/ml at 48 hrs) but was still substantial at 72 hrs post-infection (7.3×10^4^±3.8×10^4^ pfu/ml). The quantitative PCR also revealed a higher number of differentially expressed genes, with 63 genes at the 48 hr time point and 82 genes at the 72 hr time point (Table S2).

Seeking confirmation that these genes were relevant in a physiological setting, we sought to test these genes in dengue patients. Whole blood RNA samples were obtained from ten adult fever patients (age >21 years old) enrolled in the Singapore Early Dengue (EDEN) cohort study in 2005 [22]. Each was PCR diagnosed positive for either dengue serotype 1 or 3 and all showed typical dengue fever symptoms. At the second visit (∼5 days after onset of fever, onset of fever considered day 1), their platelet count had dropped below 30 (x 10^3^/µl; range 8–30, mean 20.5), and they were admitted to hospital. When these patient samples were tested for expression of the 95 selected genes by LDA, 67 genes were shown to be differentially expressed comparing blood samples taken at acute fever stage (first visit, 1–3 days after onset of fever) to convalescence (third visit, 3–4 weeks after first visit) (Table S2).

**Figure 3:**
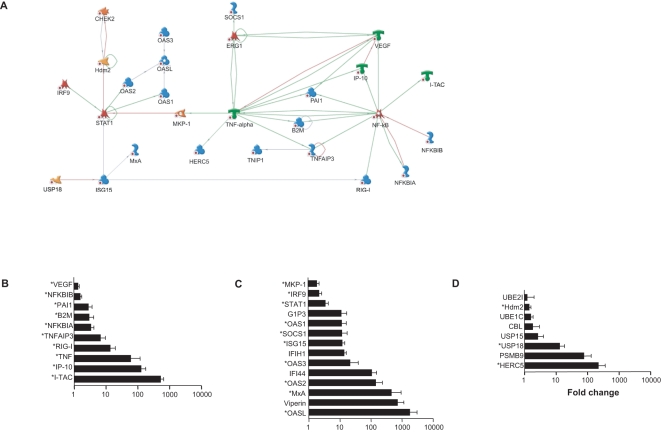
qPCR confirmed, common differentially expressed genes in HepG2, A549 cell lines and dengue patients. (A) A network of direct interactions (highlighted by * in B, C & D) was identified by the MetaCore program. Arrow head indicates directional interaction. Green are positive, red are negative, grey are neutral or unknown. Blue symbols indicate protein, green ones receptor ligand, red ones transcription factors, yellow ones proteases and orange ones kinases or phosphatases. 32 confirmed genes cluster to three biological pathways; (B) NF-κB/chemokine-genes (C) Type I IFN-mediated and (D) the ubiquitin-proteasome system. The fold increases of genes significantly differentially expressed in patients and cell lines were combined to give a pooled mean (± s.e.m.) in order to grade their relative importance.

After confirmation of the expression of certain genes in two different dengue-infected cell lines and in dengue patients, we selected those that were upregulated in at least one time point in HepG2 cells *and* in at least one time point in A549 cells *and* in dengue patient samples (comparing acute to convalescent blood samples). Fifty genes fulfilled these criteria (Table S2) and, we felt, represented common genes involved in dengue virus response, or virus replication, while excluding responses that were cell type specific. These 50 common genes were mapped by direct interactions using the MetaCore program which illustrated the close clustering and interconnectedness of a network of 29 of genes around NF-κB, TNF-α and STAT1. (Figure 3A). The NF-κB gene alone was added to the network, despite not being from our common list, to illustrate the connections between those induced by it. For example, the upregulation of IP-10, I-TAC, VEGF, PAI1, B2M, TNFAIP3 and RIG-1 could all be linked to the activation of NF-κB, while NFKB1B and NFKB1A, two feedback control genes for NF-κB activation, were also upregulated reflecting the self containment of this activation (Figure 3A). The degree of upregulation varied between the genes in this pathway with I-TAC and IP-10 being the most highly up-regulated (average expression in the two cell types and patients) genes (Figure 3B). A number of genes clustered around STAT1 were associated with the IFN pathway, and examination of the common list revealed another four genes (viperin, IFI44, IFIH1, G1P3) related to the IFN pathway that were upregulated but not mapped by the MetaCore program (Figure 3C). Finally, a number of genes related to the ubiquitin-proteasome pathway also appeared on the MetaCore map (HERC5, USP18 and Hdm2) with several more (unmapped) genes appearing on the common list (Figure 3D)

### Upregulation of chemokines IP-10 and I-TAC upon dengue infection

**Figure 4:**
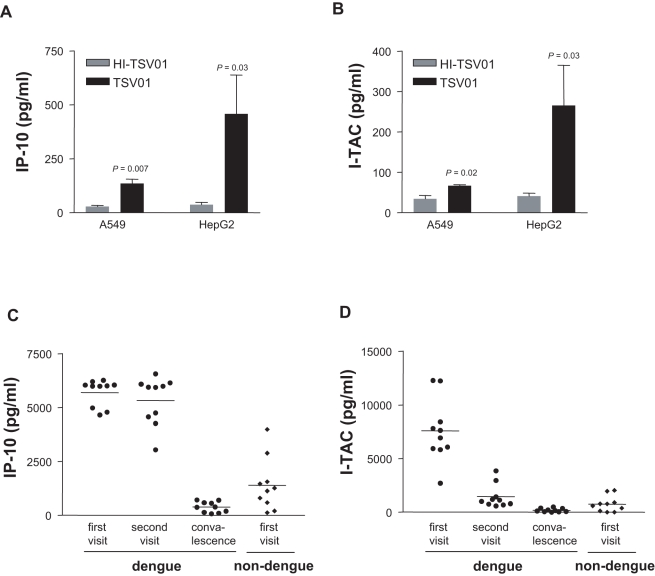
Culture supernatants of A549 and HepG2 cells infected with TSV01 (MOI 10) or heat-inactivated TSV-01 (HI-TSV01; MOI 10) for 72 hrs were analyzed for IP-10 (A) and I-TAC (B) by ELISA. Results are expressed as mean±s.e.m. where n = 3–5. Significance (P<0.05) was determined by students t-test, comparing TSV01 to HI-TSV01 at each time point. Serum samples from dengue patients (n = 10) at first visit (1–2 days after fever onset), second visit (4–5 days after fever onset), convalescence and from non-dengue fever patients (n = 10) at the first visit (1–2 days after fever onset) were assayed for the presence of IP-10 (C) and I-TAC (D) by ELISA. Results are shown as a scatter plot with a bar indicating the mean.

The two most highly upregulated common genes from the NF-κB pathway were IP-10 (or CXCL10/IFN-inducible protein 10) and I-TAC (or CXCL11/IFN-inducible I cell α chemoattractant) (Figure 3B). In order to determine if this up-regulation of gene transcription lead to translation and protein release, concentrations of IP-10 and I-TAC protein in cell culture supernatant following dengue infection were determined by ELISA. In both the A549 and HepG2 infection models, dengue infected cells produced moderate, but significant (compared to heat-inactivated virus treated cells), concentrations of IP-10 (Figure 4A) and I-TAC (Figure 4B) at 72 hrs, but not at earlier time points (data not shown). We next investigated if these, or other, NF-κB induced proteins were influencing dengue virus replication. Adding dexamethasone to the HepG2 infection model to inhibit NF-κB activation [25] prevented IP-10 and I-TAC production, but had no effect on viral replication (data not shown). These results suggest that NF-κB activation, and IP-10 and I-TAC production, do not have a direct effect on viral replication and are, rather, simply part of the immune response to infection.

Serum concentrations of IP-10 and I-TAC in the ten dengue fever patients described above, together with ten fever patients who did not have dengue (viral PCR negative) were also determined by ELISA. High concentrations of IP-10 (Figure 4C) and I-TAC (Figure 4D) were present in the serum of dengue fever patients. There was significantly more IP-10 in the serum of the patients during the first (1–2 days after fever onset) and second (4–5 days after fever onset) visits, compared to the convalescent serum (*P = *10^−15^ and *P = *10^−11^, respectively) as well as to non-dengue fever patient serum (*P* = 10^−9^ and *P* = 10^−7^, respectively). I-TAC level was also significantly higher in first visit dengue patient serum comparing to both the convalescent (*P* = 10^−7^) and non-dengue fever (*P* = 10^−6^) patients. I-TAC levels in dengue fever patients at the second visit were significantly lower than at first visit (Fig 4D).

### A strong type I IFN response and the identification of viperin as an IFN-induced anti-dengue molecule

**Figure 5:**
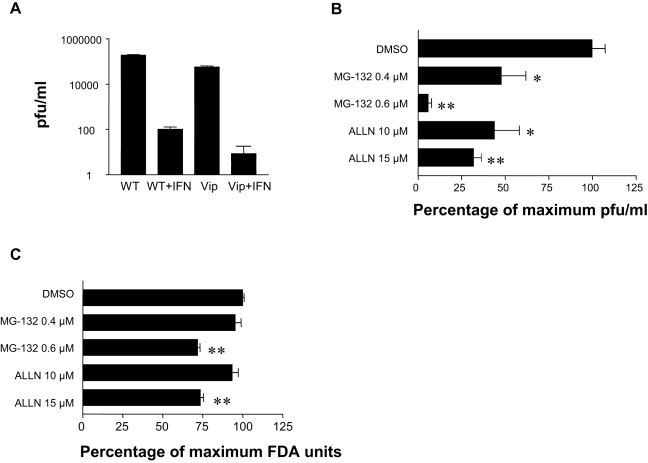
The effect of viperin overexpression and proteasome inhibitors on viral growth. (A) Wildtype (WT) and viperin overexpressing (Vip) A549 cells, with or without 12 hrs pre-treatment with IFNβ (500 U/ml), were infected with dengue virus TSV01 (MOI 1) for 48 hrs. Plaque forming units/ml in cell culture supernatant, as determined by plaque assay, are shown as mean (n = 3)±s.e.m. Significance (P<0.05) was determined by students t-test, comparing wild type A549 (WT) to viperin overexpressing cell (Vip) a P value of 0.00004 was obtained. (B) HepG2 cells were incubated with MG-132 (0.04 µM and 0.4 µM in DMSO) and ALLN (1 µM and 10 µM in DMSO) and DMSO alone for 2 hrs prior to infection with dengue virus TSV01 (MOI 10) for 48 hrs. Plaque forming units/ml in cell culture supernatant, as determined by plaque assay, are shown as mean (n = 3)±s.e.m. Significance (P<0.05) was determined by students t-test, comparing each treatment to DMSO alone. The cytotoxicity was measured in parallel using FDA (C).

We detected a large number of IFN response genes induced in both cell line infections and in dengue patients (see Figure 3C). In our study, viperin was one of the most highly upregulated genes in the type I IFN response pathway. Viperin has previously been identified as an IFN-induced anti-viral protein in HCMV and HCV infection [26],[27]. In order to investigate the role of viperin in dengue infection, we used an established A549 cell line stably overexpressing viperin (Vip) [26]. Comparing to infection in wild type A549 cells (WT), viperin overexpressing cells were significantly resistant to viral replication, as shown by plaque assay two days after infection (Figure 5A), with and without pre-treatment with IFNβ (+IFN; 500 U/ml). Although pre-treatment with IFNβ had the greater anti-viral effect, viperin over expression alone resulted in a small, but significant, reduction in virus production both with (*P* = 0.038) and without (*P* = 0.0004) IFNβ pre-treatment. These results suggest that viperin is an functional component of the IFN-mediated response to dengue, and demonstrate, for the first time, that viperin could be part of the anti-dengue response.

### Proteasome inhibitors inhibit dengue replication in the HepG2 cell line

Ubiquitination, another pathway identified in our list of common genes, is a key component of the immune system, the conjugation of single, or multiple, ubiquitin molecules to a protein targets it for defined subcellular localization, or destruction in the proteasome [28]. Components of the ubiquitin-proteasome system have been shown to be required for the maturation and release of a number of viruses (see review [29]). In order to investigate the role of ubiquitin-proteasome system in dengue infection, we introduced proteasome inhibitors, MG-132 and ALLN, to the HepG2 dengue infection model [30]. At lower concentrations, both MG-132 and ALLN significantly reduced virus replication in the cell lines by over 50% (Figure 5B). An examination of the effects of these compounds on the integrity and viability of inhibitor-treated cells using fluorescein diacetate revealed no cytotoxicity at these concentrations (Figure 5C). Higher concentrations of the inhibitors had an even greater effect (>90% reduction in pfu/ml) but with a degree of cytotoxicity (Figures 5B, 5C).

## Discussion

Despite the fact that dengue is a major disease affecting the tropical world, little is known of its pathogenesis due, partly, to the lack of a suitable animal model and the complex cell interactions in infected individuals. Using microarray analysis of gene expression and high throughput quantitative real-time PCR, we investigated the host response to dengue infection in cell lines and in DF patients. We further validated our microarray results by functional study of the identified genes. Although dendritic cells, Langerhans cells and monocytes have been proposed as the principle reservoirs of viral infection [4], it is not clear which other tissues, if any, are targeted. Immunohistochemistry and *in-situ* hybridisation studies of biopsies from DHF patients have indicated that a range of tissues are infected by the virus, including cells in the liver, spleen, lung, kidney and peripheral blood [5]. We chose the HepG2 cell line as the primary cell model for this study as it readily supported viral replication, and it is derived from the liver, which might be of some clinical relevance. The A549 cell line was included as it was also an excellent supporter of viral replication even though there is, presumably, little or no clinical relevance. The final clinical relevance came from the validation of the genes in 10 DF patients from the Singapore EDEN cohort. The 10 patients were selected based on low platelet count, because of the lack of WHO DHF/DSS manifestation in Singapore adult patients. Despite the limited sample number, and the wide range of collection times, variation between individuals at the same time point was ruled out by statistical analysis. We believe that with more patients, more genes that are differentially expressed would be identified.

The overlap of upregulated genes, determined by quantitative PCR, in the two cell systems and in patients removed any responses that were unique to the cell type. In fact, we observed cell type specific gene changes such as IL-8, PAI-1 and RANTES that were upregulated in the cell lines but not the patient samples while the anti-inflammatory cytokine IL-10 was upregulated in the patient samples but not the cell lines, indicative of the effects of multiple cell types in an *in vivo* system. Similarly, IFNβ was upregulated in the cell lines while IFNγ was in patient samples. However, it is the large overlap between the *in vitro* infection and patient samples that warrants most attention.

The two most highly upregulated chemokines were IP-10 and I-TAC. IP-10 and I-TAC are both ligands for the CXCR3 chemokine receptor and the production of these chemokines leads to the recruitment of CXCR3 expressing T cells and NK cells [31],[32]. Increased IP-10 and I-TAC expression has been seen in various viral infections, especially viral meningitis [33]. In SARS patients, elevated IP-10 early in infection was shown to be a predictor for a more severe outcome [34]. Neuronal IP-10 was shown to be involved in the recruitment of T cells in West Nile virus encephalitis [35]. Elevated I-TAC mRNA and protein has also been found in the liver of chronic Hepatitis C patients [36]. In dengue infection, various chemokines have previously been found to be induced in dengue patients, including IL-8, MIP-1α, MIP-1β, RANTES and MCP-1, but IP-10 and I-TAC have not been examined (see review [37]).

In a dengue intracerebral mouse infection models, CXCR3^−/−^ and IP10^−/−^ mice both had higher mortality rate than wild type mice after infection, indicating that IP-10 and CXCR3 receptors are part of the host defense mechanism, most likely in recruitment of T cells to the infection site [38]. IP-10 was also proposed to compete with virus binding on the receptor *in vitro* [39]. However, the relevance of this study to human patients is not clear. Although IFNγ, α/β and NF-κB could all induce the production of IP-10 and I-TAC, prevention of IP-10 and I-TAC via NF-κB inhibition clearly reduced the IP-10 level in HepG2 cells. Furthermore, IFNβ and IFNγ were not consistently upregulated in the two cell lines or in DF patients in our study (data not shown). Therefore, we believe that NF-κB activation rather than IFN induction played the major role in elevated IP-10 and I-TAC in dengue infection. The inhibition of NF-κB, and consequent IP-10 and I-TAC inhibition, had no demonstrable effect on dengue replication in cell lines, suggesting the more complex role for these chemokines in a multi-cellular *in vivo* setting. Concentrations of each chemokine were significantly higher during the early stages of dengue fever, while I-TAC levels were reduced at the second visit, IP-10 levels remained high. The persistently high level of IP-10 might also contribute to the immunopathogenesis of dengue although this would require further investigation. It may be that concentrations of IP-10 and I-TAC, in combination with a viral antigen, could be used as an early marker for dengue fever. It is interesting to note that the level of IP-10 and I-TAC were independent of previous history of dengue infection, as half of the ten tested patients were suffering from a secondary infection. The common clinical feature of the ten selected patients was the low platelet count at second visit. Because of the lack of severe dengue cases (by WHO definition) in Singapore, the low platelet count was used as the measure of severity by which the ten patients from the cohort study were classified as having more severe dengue fever. We cannot at this point make a link between the level of IP-10, I-TAC and disease severity because of the small number of cases used in this study, a more detailed clinical study, currently underway, aims to determine if the concentrations of IP-10 and I-TAC during early stages of infection are linked to the progression to more severe forms of disease.

The ability of IFN pre-treatment to inhibit subsequent dengue replication has been previously reported [9],[40], as has the importance of IFN in the anti-viral response [41]. Our results indicated that viperin was one of the most highly upregulated genes in all models following dengue infection. Viperin encodes for an IFN-inducible antiviral protein shown to be associated with the endoplasmic reticulum and redistributed to the Golgi apparatus and cytoplasmic vacuoles following human cytomegalovirus (HCMV) infection [26]. The induction of viperin has been suggested to be anti-viral in both HCMV [26] and hepatitis C (HCV) [27] infections, but this is its first association with dengue infection. The use of viperin overexpressing A549 cells demonstrated that, in addition to being significantly upregulated during infection, viperin is directly involved in the anti-viral response to dengue, as shown by the suppression of dengue replication in the presence of increased expression of viperin. This effect was not as significant as the effect of IFNβ alone suggesting that viperin is only a part of the IFN-mediated response to dengue. The molecular mechanism of the action of viperin to counter dengue infection will need to be further studied.

The ubiquitin-proteasome system is the cellular machinery involved in the conjugation of single or multiple ubiquitin molecules to direct protein trafficking or degradation (reviewed in [28]). In HCV infection, E6AP ubiquitin ligase was reported to mediate the ubiquitination and degradation of the virus core protein [42]. HCV polymerase has also been shown to interact with a ubiquitin-like protein leading to its degradation [43]. In dengue, ubiquitin-proteasome genes have not been reported to be involved in the dengue virus life cycle although a recent publication has shown that dengue envelope protein interacts with SUMO-1 conjugating enzyme 9 (Ubc9) and that overexpression of Ubc9 reduces virus production in a cell line [44].

Our TLDA results indicated that there was significant upregulation of a number of ubiquitin-proteasome system related genes during dengue virus replication but it was unclear if this response was anti-viral or if the dengue virus utilised components of the ubiquitin-proteasome system for replication. Use of proteasome inhibitors, MG-132 and ALLN, significantly reduced the release of dengue virus following infection of the HepG2 cell line. Previously, it has been shown that the effect of proteasome inhibitors may be virus specific with these compounds less (or non-) effective against virus such as influenza [45] and equine infectious anemia virus [46]. The effect of proteasome inhibition on viral replication appears to be mediated via a number of different processes including the involvement of ubiquitin or the proteasome in virus assembly [45], budding [47] and release and maturation [48]. Further studies may elucidate the exact role the ubiquitin-proteasome plays in dengue virus replication which may involve trafficking of the virus to the plasma membrane or the maturation and fusion of the virus upon release [49].

It is instructive to compare our results with those of a published microarray study investigating host responses in patient blood [50]. That study identified genes whose protein products are expressed in the ER to be the most significantly enriched, which directly overlaps with our identification of the ubiquitin pathway; specifically including UBE2 and the PSMB genes. This may represent a process fundamental to viral replication in any system. In addition, Simmons *et al.* described host responses associated with dengue shock syndrome that clearly overlap with our interferon related gene list. In particular, G1P genes, OAS genes, IFI genes and Mx genes were found in both studies, suggesting that the amount of viral replication may be directly related to clinical outcome.

Gene arrays of a number of cell systems during dengue infection revealed many genes, and host response pathways, that were upregulated during dengue infection. Further functional analyses distinguished between host response pathways involved in initiating an innate signaling response (NF-κB mediated genes and IFN pathway) and those involved in virus replication (ubiquitin-proteasome system). Specific components of the response to virus, such as viperin and IP-10 and I-TAC have been implicated in dengue infection for the first time. Further investigation of these components, together with the precise role of the ubiquitin-proteasome system in virus replication, may lead to drug targets for dengue. The use of gene array in multiple cell systems to investigate genes involved in virus replication, used in concert with functional studies, has proved to be a valid approach for discovery of novel markers and genes for understanding the host response to dengue and, ultimately, therapeutics against dengue.

## Supporting Information

Table S1 HepG2 Microarray Gene List. List of HepG2 transcripts (n = 132), identified as differentially expressed by SAM analysis, in response to dengue virus TSV01 compared to heat inactivated virus 48 and 72 hours post infection. Transcripts are placed in groups according to biological processes. qV is the SAM calculated q value for each gene (following SAM significance selection based on a Delta value calculated from the variance between the sample sets, see Material and Methods). F.C. indicates fold change. “-” represents no significant change. Genes selected for real-time PCR validation through a TaqMan low density array (TLDA) platform are indicated by a tick.
(0.28 MB DOC)

Table S2 Quantitative PCR by Taqman based low density array in HepG2, A549 and Singapore dengue fever patients. Fold increase in gene expression as determined by quantitative PCR. For HepG2 and A549, the fold change was calculated based on dengue virus infection over heat-inactivated virus infection. For dengue fever patients (Patients), the fold change was calculated using patient blood samples collected at the first visit (∼1–2 days after onset of fever) over the convalescence (3–4 weeks after the acute fever). Upregulation is shown in black and down-regulation in red. “1.0” represents no significant change (significance determined by q value <5, see Material and Method). Genes that were significantly up-regulated in at least one time point in HepG2 and in at least one time point in A549 and in Patients are indicated with P-values, calculated by standard student T test and selected based on a cut off at P<0.05.
(0.19 MB DOC)
