# Supplementary material for: Host Gene Expression Profiling of Dengue Virus Infection in Cell Lines and Patients
Source: PLoS Negl Trop Dis. 2007 Nov 21;1(2):e86. doi: 10.1371/journal.pntd.0000086 (PMC2100376; doi:10.1371/journal.pntd.0000086)
Supplement: Table S1 — HepG2 Microarray Gene List. List of HepG2 transcripts (n = 132), identified as differentially expressed by SAM analysis, in response to dengue virus TSV01 compared to heat inactivated virus 48 and 72 hours post infection. Transcripts are placed in groups according to biological processes. qV is the SAM calculated q value for each gene (following SAM significance selection based on a Delta value calculated from the variance between the sample sets, see Material and Methods). F.C. indicates fold change. “-” represents no significant change. Genes selected for real-time PCR validation through a TaqMan low density array (TLDA) platform are indicated by a tick. (0.28 MB DOC) [file pntd.0000086.s001.doc]

| **Supplementary Table 1** | |  | | | | | | |
| --- | --- | --- | --- | --- | --- | --- | --- | --- |
| **Gene Name** | **Transcript** | **Group** | **Gene description** | **48 Hours** | | **72 Hours** | | **TLDA** |
|  |  |  |  | **qV** | **F.C.** | **qV** | **F.C.** |  |
| CCL4 | NM_002984 | NFkB related | Cytokine and chemokine mediated signaling pathway; | 10.9 | 1.6 | 3.0 | 2.2 | ✓ |
| CCL5 | NM_002985 | NFkB related | Cytokine and chemokine mediated signaling pathway; | 3.8 | 2.5 | 3.0 | 2.5 | ✓ |
| IP10 | NM_001565 | NFkB related | Cytokine and chemokine signaling;Macrophage-mediated immunity | 3.8 | 2.0 | 7.0 | 3.4 | ✓ |
| I-TAC | NM_005409 | NFkB related | Cytokine and chemokine signaling;Macrophage-mediated immunity | 5.4 | 1.8 | 7.0 | 2.8 | ✓ |
| IL11b | NM_000881 | NFkB related | interleukin 11 (IL11b) mRNA |  |  | 7.0 | 1.7 | ✓ |
| IL8 | M17017 | NFkB related | Chemokine activity, attracts neutrophils, basophils, and t-cells | 3.8 | 4.4 | 3.0 | 6.6 | ✓ |
| NFKBIA | NM_020529 | NFkB related | mRNA transcription;NF-kappaB cascade;Intracellular protein traffic | 10.9 | 1.7 | 3.0 | 2.5 | ✓ |
| NFKBIB | NM_002503 | NFkB related | mRNA transcription;NF-kappaB cascade;Intracellular protein traffic | - | - | 23.1 | 1.4 | ✓ |
| TNFAIP | NM_006290 | NFkB related | tumor necrosis factor, alpha-induced protein 3 (TNFAIP3) mRNA | - | - | 12.3 | 1.9 | ✓ |
| ATF3 | NM_004024 | Interferon related | mRNA transcription regulation;Induction of apoptosis | 5.4 | 2.1 | 4.1 | 3.3 | ✓ |
| G1P2 | NM_005101 | Interferon related | Proteolysis, interferon-stimulated protein, 15 kDa (ISG15) | 3.8 | 11.3 | 3.0 | 9.2 | ✓ |
| G1P3 | NM_002038 | Interferon related | interferon, alpha-inducible protein (clone IFI-6-16) | 3.8 | 2.6 | 3.0 | 2.3 | ✓ |
| IFI44 | NM_006417 | Interferon related | interferon-induced, hepatitis C-associated microtubular aggregate protein | - | - | 3.0 | 2.0 | ✓ |
| IFIT1 | NM_001548 | Interferon related | interferon-induced protein with tetratricopeptide repeats 1;IFIT1 | 3.8 | 4.3 | 3.0 | 5.8 |  |
| IFIT2 | AF026944 | Interferon related | Interferon-induced protein with tetratricopeptide repeats 2 | - | - | 7.0 | 2.6 |  |
| IFIT3-1 | AF026943 | Interferon related | Interferon-induced protein with tetratricopeptide repeats 3 | 3.8 | 5.5 | 3.0 | 7.9 | ✓ |
| IFIT3-2 | NM_001549 | Interferon related | interferon-induced protein with tetratricopeptide repeats 3 | 3.8 | 2.6 | 3.0 | 2.6 |  |
| IFNB1 | NM_002176 | Interferon related | interferon, beta 1, fibroblast | 3.8 | 3.2 | 3.0 | 3.1 | ✓ |
| ISGF3G | NM_006084 | Interferon related | mRNA transcription regulation;Interferon-mediated immunity | 5.4 | 2.0 | 12.3 | 1.7 | ✓ |
| MX1 | NM_002462 | Interferon related | myxovirus (influenza virus) resistance 1, interferon-inducible protein p78 | 3.8 | 7.3 | 3.0 | 5.0 | ✓ |
| OAS1 | NM_016816 | Interferon related | nucleotide and nucleic acid metabolism;Interferon-mediated immunity | 3.8 | 4.4 | 3.0 | 3.6 | ✓ |
| OAS2 | NM_002535 | Interferon related | nucleotide and nucleic acid metabolism;Interferon-mediated immunity | 3.8 | 5.6 | 3.0 | 4.0 | ✓ |
| OAS3 | NM_006187 | Interferon related | Nucleic acid binding;Nucleotidyltransferase;Defense/immunity protein | - | - | 23.1 | 1.4 | ✓ |
| OASL | AF063611 | Interferon related | 2'-5'oligoadenylate synthetase-related protein p56 (OASL) | 10.9 | 1.7 | 3.0 | 2.1 | ✓ |
| VIPERIN-1 | AF026941 | Interferon related | Virus inhibitory, endoplasmic reticulum-associated, interferon inducible | 3.8 | 3.4 | 3.0 | 5.0 | ✓ |
| VIPERIN-2 | AF026942 | Interferon related | Virus inhibitory, endoplasmic reticulum-associated, interferon inducible | 3.8 | 3.8 | 3.0 | 3.9 |  |
| IER3 | NM_003897 | Interferon related | immediate early response 3 | - | - | 12.3 | 2.0 | ✓ |
| IFIH | AL080107 | Interferon related | RNA helicase, DEAD box protein, upregulated with beta-interferon | 3.8 | 2.1 | 23.1 | 1.9 |  |
| IFIT5 | NM_012420 | Interferon related | retinoic acid- and interferon-inducible protein (58kD) (RI58) | 10.9 | 1.6 | - | - |  |
| IFRG28 | AJ251832 | Interferon related | 28kD interferon responsive protein | 3.8 | 1.9 | 4.1 | 2.2 | ✓ |
| MDA5 | AF095844 | Interferon related | interferon induced with helicase C domain 1;IFIH1 | 3.8 | 4.7 | 3.0 | 7.0 | ✓ |
| SP110 | NM_004510 | Interferon related | interferon-induced protein 75, 52kD (IFI75) mRNA transcription | 3.8 | 2.0 | - | - |  |
| STAT1-1 | AK022231 | Interferon related | signal transducer and activator of transcription | 3.8 | 1.8 | - | - |  |
| STAT1-2 | NM_007315 | Interferon related | signal transducer and activator of transcription | 3.8 | 2.9 | 3.0 | 2.2 | ✓ |
| BIRC3 | NM_001165 | Ubiquitin related | Ubiquitin-protein ligase activity, Anti-apoptosis | - | - | 4.1 | 2.0 |  |
| C17orf27 | AB046774 | Ubiquitin related | Ubiquitin-protein ligase activity | 5.4 | 1.8 | - | - |  |
| DTX3L | AK025135 | Ubiquitin related | Ubiquitin-protein ligase activity | 5.4 | 1.8 | 23.1 | 1.5 |  |
| HERC5 | NM_016323 | Ubiquitin related | Ubiquitin-protein ligase, cyclin-E binding protein 1 | 3.8 | 6.0 | 3.0 | 10.6 | ✓ |
| HERC6 | NM_017912 | Ubiquitin related | Ubiquitin-protein ligase | - | - | 23.1 | 1.6 |  |
| PSMB9 | NM_002800 | Ubiquitin related | proteasome (prosome, macropain) subunit, beta type, 9 | 7.7 | 1.8 | 4.1 | 1.7 | ✓ |
| RNF36 | AL360161 | Ubiquitin related | Ubiquitin-protein ligase activity, apoptosis | 5.4 | 1.7 | - | - |  |
| UBE2L6 | NM_004223 | Ubiquitin related | ubiquitin-conjugating enzyme E2L 6, ligase | 7.7 | 1.8 | 12.3 | 1.5 | ✓ |
| USP15 | AF106069 | Ubiquitin related | Cysteine protease deubiquitinating enzyme | 3.8 | 2.3 | 23.1 | 1.7 | ✓ |
| USP18 | NM_017414 | Ubiquitin related | Cysteine protease, ubiquitin specific protease 18 | 3.8 | 2.4 | 4.1 | 1.9 | ✓ |
| AB037725 | AB037725 | SAM selected | KIAA1304 | - | - | 23.1 | 1.5 |  |
| AF086367 | AF086367 | SAM selected | ZD66F04 | - | - | 7.0 | 1.7 |  |
| AGR2 | NM_006408 | SAM selected | anterior gradient 2 | - | - | 3.0 | 2.1 |  |
| AK000877 | AK000877 | SAM selected | AJ002784 | - | - | 3.0 | 2.8 |  |
| AK021733 | AK021733 | SAM selected | HEMBA1004730 | 10.9 | 1.7 | - | - |  |
| AK021936 | AK021936 | SAM selected | HEMBA1007073 | - | - | 7.0 | 1.7 |  |
| AL049423 | AL049423 | SAM selected | AL049423 | - | - | 4.1 | 1.7 |  |
| AL110204 | AL110204 | SAM selected | AL110204 | - | - | 12.3 | 1.6 |  |
| ATP6B1 | NM_001692 | SAM selected | nucleotide and nucleic acid transport;Cation transport | - | - | 23.1 | 1.5 | ✓ |
| B2M | NM_004048 | SAM selected | MHCI-mediated immunity | - | - | 4.1 | 1.7 | ✓ |
| BG610654 | AK000422 | SAM selected | Beta-galactosidase | - | - | 12.3 | 1.6 |  |
| BHLHB2 | NM_003670 | SAM selected | mRNA transcription regulation;Cell proliferation and differentiation | - | - | 12.3 | 1.8 |  |
| BST2 | NM_004335 | SAM selected | bone marrow stromal cell antigen | 12.8 | 1.7 | 23.1 | 1.5 |  |
| BTBD2 | NM_017797 | SAM selected | protein-protein interactions, cytoplasmic bodies | - | - | 12.3 | 1.6 |  |
| BTG3 | NM_006806 | SAM selected | Cell proliferation and differentiation | - | - | 4.1 | 1.7 | ✓ |
| C14orf161 | AK024360 | SAM selected | None | - | - | 7.0 | 1.9 |  |
| C15ORF2 | NM_018958 | SAM selected | cell survival, calcium- sequestering | - | - | 12.3 | 2.0 |  |
| CBFA1 | AF053952 | SAM selected | OSF2 transcription factor | - | - | 7.0 | 1.8 |  |
| CHEK2 | NM_007194 | SAM selected | Protein phosphorylation;Stress response | - | - | 4.1 | 1.8 | ✓ |
| CITED2 | NM_006079 | SAM selected | Transcription cofactor | - | - | 23.1 | 1.4 | ✓ |
| CNN1 | NM_001299 | SAM selected | Muscle contraction | - | - | 23.1 | 1.6 | ✓ |
| D17210 | D17210 | SAM selected | hmd3f02m3 | - | - | 12.3 | 1.6 |  |
| DAAM1 | AB014566 | SAM selected | Non-motor actin binding protein | - | - | 23.1 | 1.5 |  |
| DDX58 | NM_014314 | SAM selected | Nucleoside, nucleotide and nucleic acid metabolism;Apoptosis | 3.8 | 2.2 | 7.0 | 1.8 | ✓ |
| DUSP1(1) | AJ227912 | SAM selected | Response to oxidative stress | - | - | 3.0 | 1.9 |  |
| DUSP1(2) | NM_004417 | SAM selected | Response to oxidative stress | - | - | 3.0 | 1.9 | ✓ |
| EGR1 | NM_001964 | SAM selected | mRNA transcription regulation | - | - | 7.0 | 1.9 | ✓ |
| ENSest7951 | ENSest7951 | SAM selected | ENSestG00000007951 | - | - | 7.0 | 1.7 |  |
| FIP2 | AF061034 | SAM selected | TNFA or Fas-ligand pathways (apoptosis, inflammation, vasoconstriction) | - | - | 23.1 | 1.4 |  |
| FLJ11021 | AK001883 | SAM selected | splicing factor, arginine/serine-rich 4 | - | - | 23.1 | 1.5 |  |
| FLJ20035 | AK001649 | SAM selected | Helicase activity, Nucleic acid binding | - | - | 12.3 | 1.6 |  |
| FLJ20156 | NM_017691 | SAM selected | Leucine rich repeat | 10.9 | 1.6 | 7.0 | 2.3 |  |
| FLJ22761 | AK026414 | SAM selected | Hexokinase-1 | - | - | 23.1 | 2.3 |  |
| FLJ34585 | AK022228 | SAM selected | None | - | - | 23.1 | 1.5 |  |
| FOS(1) | NM_005252 | SAM selected | Viral oncogene, mRNA transcription regulation;Immunity and defense; | - | - | 3.0 | 2.4 |  |
| FOS(2) | NM_005252 | SAM selected | Viral oncogene, mRNA transcription regulation;Immunity and defense; | - | - | 4.1 | 2.0 |  |
| GADD45A | NM_001924 | SAM selected | DNA repair;Stress response;Apoptosis;Cell cycle control | - | - | 12.3 | 1.6 | ✓ |
| GEM | NM_005261 | SAM selected | G-protein mediated signaling;Cell structure and motility | - | - | 3.0 | 2.0 | ✓ |
| GENX-3414 | NM_003943 | SAM selected | Genethonin-1, Carbohydrate metabolism | - | - | 7.0 | 1.5 | ✓ |
| GHRGV9A | AF230800 | SAM selected | growth hormone receptor gene, 5'UTR V9A region | 10.9 | 2.0 | - | - |  |
| H1F2 | NM_005319 | SAM selected | Chromatin packaging and remodeling | - | - | 7.0 | 1.6 | ✓ |
| HEY1 | NM_012258 | SAM selected | Basic helix-loop-helix transcription factor;Nucleic acid binding | - | - | 3.0 | 1.9 | ✓ |
| HSP70B | NM_002155 | SAM selected | Protein folding;Protein complex assembly;Stress response | - | - | 12.3 | 1.7 | ✓ |
| HSPA1B | NM_005346 | SAM selected | Protein folding;Protein complex assembly;Stress response | - | - | 12.3 | 2.0 | ✓ |
| HSPF1 | NM_006145 | SAM selected | Protein folding | - | - | 4.1 | 1.8 | ✓ |
| IER5 | NM_016545 | SAM selected | immediate early response 5, cellular response to mitogenic signals | - | - | 4.1 | 1.7 |  |
| IGFBP6 | M62402 | SAM selected | Human insulin-like growth factor binding protein 6 | - | - | 12.3 | 1.6 | ✓ |
| ITGB3 | NM_000212 | SAM selected | Cell adhesion-mediated signaling;Blood clotting;Cell motility | - | - | 23.1 | 1.5 | ✓ |
| JUN(1) | NM_002228 | SAM selected | mRNA transcription regulation;JNK cascade;Cell cycle control | - | - | 3.0 | 1.8 |  |
| JUN(2) | NM_002228 | SAM selected | mRNA transcription regulation;JNK cascade;Cell cycle control | - | - | 23.1 | 1.6 |  |
| KIAA0590 | AK023912 | SAM selected | Collagen alpha-1(I) chain precursor | - | - | 23.1 | 1.4 |  |
| KIAA1404 | AK023836 | SAM selected | Transcription factor activity | - | - | 23.1 | 1.8 |  |
| KLF6(1) | AL117595 | SAM selected | Transcriptional activator activity, B-cell growth and development | - | - | 23.1 | 1.5 |  |
| KLF6(2) | NM_001300 | SAM selected | Transcriptional activator activity, B-cell growth and development | - | - | 23.1 | 1.6 |  |
| KRT17 | NM_000422 | SAM selected | Intermediate filament;Structural protein | 5.4 | 1.9 | 3.0 | 3.8 | ✓ |
| KYNU | NM_003937 | SAM selected | Amino acid metabolism | - | - | 23.1 | 1.7 | ✓ |
| LBA1 | AB002340 | SAM selected | belongs to the ribosomal protein s12p family | - | - | 7.0 | 1.7 |  |
| LGP2 | AK021416 | SAM selected | Nucleoside, nucleotide and nucleic acid metabolism | 3.8 | 2.6 | 3.0 | 2.6 |  |
| LOC283737 | AL133446 | SAM selected | lysosome, degradation of dermatan and keratan sulfates. | - | - | 23.1 | 1.5 |  |
| LOC93082 | AL389981 | SAM selected | ortholog of mouse lung-inducible C3HC4 RING domain protein | 5.4 | 2.3 | 7.0 | 5.1 |  |
| LPIN1 | D80010 | SAM selected | Lipid metabolism;Developmental processes | - | - | 4.1 | 1.9 |  |
| LRRN3 | AL442092 | SAM selected | Leucine rich repeat neuronal 3 | - | - | 7.0 | 1.6 |  |
| LY6E(1) | NM_002346 | SAM selected | lymphocyte antigen 6 complex, locus E;LY6E | 5.4 | 2.4 | 3.0 | 2.1 |  |
| LY6E(2) | NM_002346 | SAM selected | lymphocyte antigen 6 complex, locus E;LY6E | 5.4 | 1.9 | 12.3 | 1.8 |  |
| MGC40405 | AB046797 | SAM selected | Zinc finger, SWIM-type containing 6 | - | - | 23.1 | 1.6 |  |
| MGC45731 | AK027019 | SAM selected | Zinc finger protein 697, transcription factor | - | - | 7.0 | 1.7 |  |
| PARP14 | AB033094 | SAM selected | Protein amino acid ADP-ribosylation | 5.4 | 1.9 | 4.1 | 2.2 |  |
| PLK2 | NM_006622 | SAM selected | Protein phosphorylation;Intracellular signaling cascade | - | - | 12.3 | 1.5 | ✓ |
| pLSB8 | U03241 | SAM selected | Clone pLSB8 chromosome 21 STS | - | - | 23.1 | 1.7 |  |
| PMAIP1 | D90070 | SAM selected | Oncogenesis | - | - | 3.0 | 2.8 |  |
| PTTG1 | NM_004219 | SAM selected | DNA repair;mRNA transcription regulation;Cell cycle control | - | - | 23.1 | 1.6 |  |
| RAB27A | NM_004580 | SAM selected | Receptor mediated endocytosis;General vesicle transport | - | - | 23.1 | 1.4 | ✓ |
| REC8L1 | NM_005132 | SAM selected | chromatid cohesion phosphoprotein of the rad21p family | 18.8 | 1.5 | - | - |  |
| RGS2 | NM_002923 | SAM selected | G-protein mediated signaling | - | - | 12.3 | 1.7 | ✓ |
| RND1 | NM_014470 | SAM selected | G-protein mediated signaling;Cell structure and motility | - | - | 12.3 | 2.0 | ✓ |
| RRAD | NM_004165 | SAM selected | GTPase activity TAS GOA/IPI | 3.8 | 3.0 | 7.0 | 1.9 | ✓ |
| SDCBP | NM_005625 | SAM selected | GTPase mediated signal transduction | - | - | 23.1 | 1.5 |  |
| SHB | NM_003028 | SAM selected | SHB adaptor protein (a Src homology 2 protein) (SHB) | - | - | 12.3 | 1.5 |  |
| SPAG9 | NM_003971 | SAM selected | Intracellular signaling cascade;Transport;Cell structure and motility | - | - | 12.3 | 1.6 |  |
| SUMO2 | NM_006937 | SAM selected | Protein modification;Inhibition of apoptosis | - | - | 7.0 | 1.9 |  |
| TES | NM_015641 | SAM selected | Actin binding cytoskeletal protein;Structural protein | - | - | 23.1 | 1.4 |  |
| TNIP1 | NM_006058 | SAM selected | Nef-associated factor 1 | - | - | 12.3 | 1.6 | ✓ |
| TOP1 | J03250 | SAM selected | DNA unwinding | - | - | 12.3 | 1.8 |  |
| TSPYL2 | AF273046 | SAM selected | DNA replication;Chromatin packaging and remodeling;Apoptosis | - | - | 23.1 | 1.7 |  |
| TULP3 | NM_003324 | SAM selected | tubby like protein 3 | - | - | 4.1 | 1.7 | ✓ |
| VIP | NM_003381 | SAM selected | Regulation of vasoconstriction, dilation | - | - | 12.3 | 1.9 | ✓ |
| WBP5 | NM_016303 | SAM selected | Transcription factor | - | - | 7.0 | 1.5 |  |
| ZC3HAV1 | NM_020119 | SAM selected | Zinc finger CCCH type antiviral protein 1 | - | - | 12.3 | 1.7 |  |
