# Supplementary figures and images for: Host Gene Expression Profiling of Dengue Virus Infection in Cell Lines and Patients
Source: PLoS Negl Trop Dis. 2007 Nov 21;1(2):e86. doi: 10.1371/journal.pntd.0000086 (PMC2100376; doi:10.1371/journal.pntd.0000086)

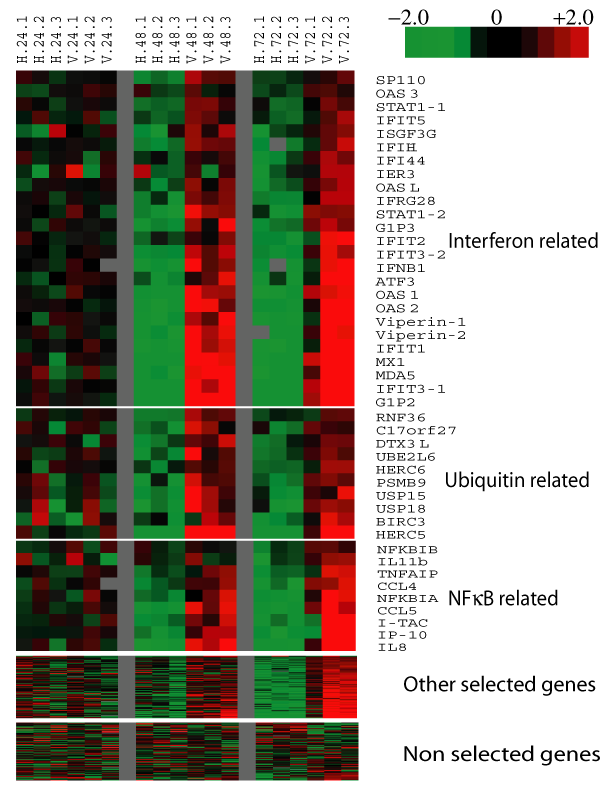

Supplement: Table S2 — Quantitative PCR by Taqman based low density array in HepG2, A549 and Singapore dengue fever patients. Fold increase in gene expression as determined by quantitative PCR. For HepG2 and A549, the fold change was calculated based on dengue virus infection over heat-inactivated virus infection. For dengue fever patients (Patients), the fold change was calculated using patient blood samples collected at the first visit (∼1–2 days after onset of fever) over the convalescence (3–4 weeks after the acute fever). Upregulation is shown in black and down-regulation in red. “1.0” represents no significant change (significance determined by q value <5, see Material and Method). Genes that were significantly up-regulated in at least one time point in HepG2 and in at least one time point in A549 and in Patients are indicated with P-values, calculated by standard student T test and selected based on a cut off at P<0.05. (0.19 MB DOC) [file pntd.0000086.s002.doc]
